# EARLY ORAL RE-FEEDING ON ONCOLOGY PATIENTS SUBMITTED TO GASTRECTOMY FOR GASTRIC CANCER

**DOI:** 10.1590/S0102-67202015000300014

**Published:** 2015

**Authors:** Andressa Madalozo LAFFITTE, Camila Brandão POLAKOWSKI, Massakazu KATO

**Affiliations:** From Hospital Erasto Gaertner (Erasto Gaertner Hospital), Curitiba, PR, Brazil

**Keywords:** Gastrectomy, Neoplasia, Diet

## Abstract

**Background:**

There is no advantage in maintaining patients on oral fasting after gastrointestinal elective resection. The early feeding up to 48 h can be beneficial, because it reduces infectious complications and hospital stay.

**Aim:**

Evaluate the evolution and tolerance of early oral diet in postoperative period after gastrectomy for gastric cancer.

**Methods:**

Anthropometric assessment was performed on the day of surgery, weight, height, body mass index and weight loss were measured. Acceptance of diet was evaluated as food intake (amount accepted) and gastrointestinal symptoms such as nausea, vomiting, constipation, diarrhea, abdominal distension, postoperative complications and hospital stay.

**Results:**

The sample consisted of 23 patients, 17 with partial gastrectomy and six with total gastrectomy. In the assessment of nutritional status 9% were malnourished, 54.5% normal weight, 9% overweight and 27.2% obese, but 54% had weight loss. There was good acceptance of the diet in 96,9% of the sample. Nausea and abdominal distension were present in 4,3% and 65.2% constipation. Surgical complications according to the Clavien scalle, 13% had grade V, 4.3% grade IIIA, 8.7% grade I and 73% did not have complications. The length of hospital stay was 5±2.2 days.

**Conclusion:**

Early postoperative re-feeding in total and partial gastrectomy was well tolerated by patients.

## Abstract (pt)

**Racional:**

Estudos mostram que não há vantagem em manter pacientes em jejum via oral após a ressecção eletiva gastrointestinal. A alimentação precoce até 48 h pode ser benéfica, pois diminui complicações infecciosas e permanência hospitalar.

**Objetivo:**

Avaliar a evolução e tolerância da dieta precoce via oral nos pacientes no pós-operatório de gastrectomia por câncer gástrico.

**Método:**

Foi realizada avaliação antropométrica no dia da operação aferindo peso, altura, índice de massa corporal e perda ponderal. A aceitação da dieta foi avaliada conforme ingestão alimentar (volume aceito) e sintomas gastrointestinais como náuseas, vômitos, obstipação, diarreia, distensão abdominal, complicações no pós-operatório e tempo de permanência hospilatar.

**Resultados:**

A amostra foi composta por 23 pacientes, sendo 17 com gastrectomia parcial e seis com total. A avaliação do estado nutricional demonstrou desnutrição em 9%, eutróficos em 54,5%, com sobrepeso 9% e com obesidade 27,2%, porém 54% apresentaram perda ponderal. Houve boa aceitação da dieta em 96,9% da amostra. Dos sintomas avaliados 4,3% apresentarem náuseas e distensão abdominal e 65,2% de obstipação. Das complicações cirúrgicas, conforme a escala de Clavien, 13% apresentaram complicações grau V, 4,3% grau IIIA, 8,7% grau I e 73% não apresentaram complicações. O tempo de permanência hospitalar foi de 5±2,2 dias.

**Conclusão:**

Realimentação precoce no pós-operatório de gastrectomia total e parcial foi bem tolerada pelos pacientes.

## INTRODUCTION

Stomach cancer is the fourth most incident in Brazil for men and the fifth for women 18 , except for skin cancer. It occurs mostly in men and about 65% are over 50 years 16 .

For gastric cancer the most effective treatment is surgery 27 28 . The patient underwent surgery, which removes or reduces gastric camera, can display physiological changes represented by dumping syndrome, lower protein digestion, reduced absorption of vitamin B12 and intestinal malabsorption. These changes contribute to the progressive deterioration of the nutritional status and may lead to malnutrition 3 17 26 28 .

Patients undergoing gastric resection are generally able to initiate the oral intake from three to seven days postoperatively 28 , and it is common to have prolonged fasting in abdominal operations. Gastric decompression is often used for routine prevention of ileus, nausea and vomiting after surgery and potential anastomoses protection 25 , the assumption that bowel rest must occur to ensure the healing of digestive anastomoses with less risk 1 .

Studies show, however, that there is no advantage in maintaining patients on oral fasting after the gastrointestinal elective resection. The early feeding up to 48 h can be beneficial because it reduces infectious complications and hospital stay 21 , and contributes to the healing of the anastomosis, faster recovery and is not related to complications after surgery 25 . Prolonged lack of food in the intestinal lumen generates mucosal atrophy, which may disrupt the intestinal barrier and promote bacterial translocation. When late, it may exacerbate the metabolic response to surgical trauma, with negative consequences for the nutritional status. The recommendation for feedback postoperatively for certain operations would be early onset (within 48 h) 21 .

The operations with presence of gastrointestinal anastomoses, enteroenteric, enterocolic or colorectal can start a liquid diet routine on the first day after surgery. Oliveira study showed that 74% (n=14) of the patients tolerated the volume of 300 ml by liquid diet meal 1 2 7 25 .

The objective of this study was to evaluate the progress and tolerance the start of early oral diet (up to 48 h) in patients in the postoperative period of total and partial gastrectomy demonstrating its applicability in hospital practice

## METHODS

This study was submitted to the Research Ethics Committee of the institution being approved in accordance with the Protocol. 2270, 2013. It is a prospective, descriptive and transversal study. The research was conducted between the months July to December 2013 involving 41 patients of the Abdominal Surgery Department of Erastus Gaertner Hospital, Curitiba, PR, Brazil. The sample was of patients who underwent partial or total gastrectomy in the research period. Patients unable to perform anthropometric and with late-onset diet (more than 48 h) were excluded.

**FIGURE 1:**
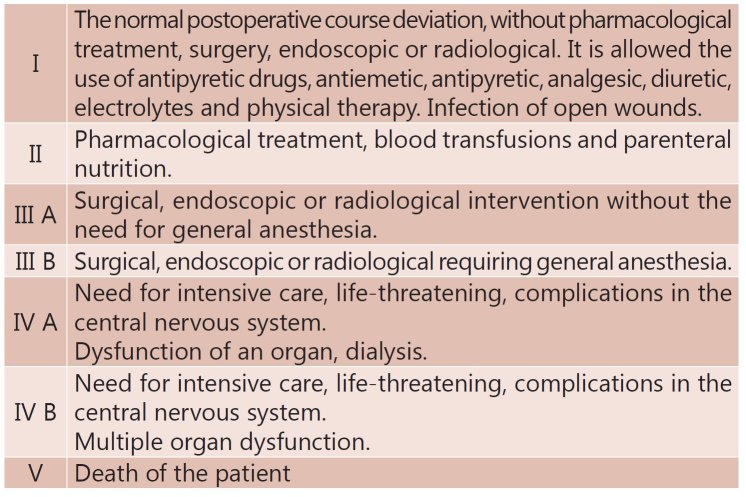
Clavien's Scale 11

The evaluation was conducted in three stages rising from the following data: 1) the day of the surgical procedure was checked for weight, height, body mass index (BMI), usual weight reported by the patient and the time lost weight (one week to six months) when compared to the current, percentage of weight loss (% WLP) involuntary dividing the sample into two groups, adults (less than 60 years) and seniors; 2) on the first day of early oral feeding (2^nd^ day after surgery, because on the 1^st^ day were fasted) when they were asked about the acceptance and tolerance of the diet by volume ingested per meal and symptoms (nausea, bloating, vomiting, diarrhea and constipation), until hospital discharge; 3) up to 30 days after surgery to evaluate possible complications (fistula, dehiscence, pneumonia, deep vein thrombosis, respiratory failure, wound infection and evaluated the degree of complications according to Clavien scale (2009), which classifies surgical complications within seven classes ( Figure 1 ).

**FIGURE 2:**
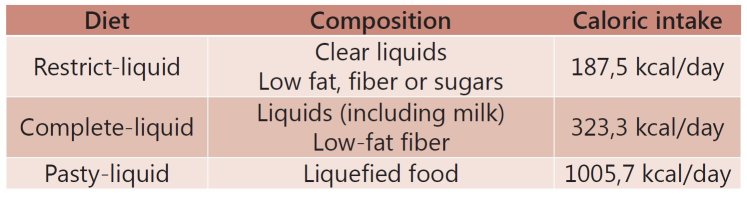
Protocol diets features for total and partial gastrectomy

The first day of diet began on the second day after surgery with retrict-liquid diet, with a volume of 700 ml fractionated into seven meals; on the second day of diet with the complete-liquid diet, with a volume of 1100ml fractionated into seven meals and on the third day of diet with pasty-liquid diet, with a volume of 1450 ml fractionated into seven meals ( Figure 2 ), standing for the fourth till seventh day of diet and progressing to soft diet for 30 days, continuing to follow the diet in the nutrition clinic. This evolution occurred through some criteria as: well acceptance (greater than 75%) of the volume offered and no vomiting. Re-admissions postoperatively added to the data collection form.

The nutritional status was calculated by BMI (BMI=weight (kg)/height (m²) and classified by the World Health Organization 8 for adult and elderly. In %PP was calculated using the formula: %PP=[(Usual P - Current P) x 100]/ usual PA. It was related the result of the percentage of weight loss and the time of such loss, based on the weight loss of 1-2% in a week, 5% in one month, 7.5% in three months and 10% in six months.

The data were tabulated in Microsoft Excel® 2007 version, with descriptive analysis (frequency, percentage) and used software used was the SPSS 19.0.

## RESULTS

In the six-month period was included 41 patients, 18 were excluded because they realized other concomitant surgical procedures (gastroenterostomy, esophagogastrectomy, jejunostomy and laparotomy), or because they started late diet, totaling 23 patients in the final sample. The age ranged from 48 to 84 years (mean 61 and median 60), in both genders. Seventeen (73.9%) underwent partial gastrectomy, eight with lymphadenectomy, and six (26.1%) to total gastrectomy with lymphadenectomy. According to clinical or pathological staging of the tumor, it was noted that almost half of the patients were in III ( Table 1 ).

**TABLE 1:** Tumor staging (n=23)

Staging	% (n)
I	17,4% (n=4)
II	8,7% (n=2)
III	43,4% (n=10)
IV	17,4% (n=4)
Gist	4,3% (n=1)
Impossible to be staged	8,7% (n=2)

To evaluate nutritional status patients were divided into adults and the elderly. In adults 9% (n=1) of patients were malnourished, 54.5% (n=6) in eutrophic, 9% (n=1) were overweight and 27.2% (n=3) obesity, however, another data evaluated was the percentage of weight loss, which found that 54.5% (n=6) of adults have had this weight loss, with 27.2% (n=3) in a serious way. In the elderly 41.6% (n=5) of patients were underweight, 41.6% (n=5) in eutrophic and 16.6% (n=2) overweight according to body mass index (BMI). Regarding weight loss 100% (n=12) showed that loss considering 58.3% were severely affected.

In assessing the evolution and acceptance of the diet protocol there was good acceptance in 95.6% on the restrict-liquid diet on the 2^nd^ postoperative period (1^st^ day of diet); 95.2% in the complete-liquid diet on 3^rd^ postoperative period (2^nd^ day of diet) and 100% in the pasty-liquid diet on the 4^th^ postoperative period (3^rd^ day of diet). Overall there was good acceptance of the diet (average 96.9%) at all stages.

When analyzing the symptoms after starting the diet, no patients presented vomiting or diarrhea in the relocation period, 4.3% had nausea and/or abdominal distension and 65,2 had constipation. The length of stay ranged from 3 to 14 days (average 5±2.2). Complications within 30 days of postoperative fistulas were 13% (n=3) and 17.2% (n=4) other complications (bronchospasm, sepsis, evisceration and vomiting). There were no other researched complications such as dehiscence, pneumonia, stroke, respiratory failure and wound infection.

In assessing the degree of postoperative complications according to Clavien's Scale, it was noted that not all grades were present in the sample. They were reported in grade I8.7%, grade III A 4.3%, grade V 13% and without complications 73.9%.

The main complication, if compared with nutritional status, was the presence of fistula. Of the 13% (n=3) found 8.6% (n=2) were elderly with low weight and 4.3% (n=1) adults with some degree of obesity and mild weight loss. Among found fistulas, 8.6% (n=2) were in the esophagojejunal anastomosis and 4.3% (n=1) in duodenal stump.

## DISCUSSION

In this study it is clear that the majority of patients (60.8%, n=14) was admitted with advanced disease according to clinical staging or pathological TNM 15 28 . Only 26% were underweight according to BMI for age, being almost half the expected according to the Brazilian Hospital Nutrition Examination Survey (IBRANUTRI); however, it should take into account that 78.3% of patients had weight loss. This item (percentage of weight loss) was not evaluated in the Murphy's study, however it concludes that the BMI alone is a poor indicator for gastrectomy for gastric cancer, where the malnutrition rate was only 12% of their sample 4 5 6 10 13 19 20 22 23 25 .

The displayed weight loss can be justified by food intake presented by patients according to tumor location. According IBNO (survey conducted in 16 Brazilian states and the Federal District) 6.5% (n=13) of patients with gastric cancer increased food intake, 71.7% (n=142) decreased and 21.7% (n=43) remained preserved. Another information weight related referred to the weight loss at 60.1% (n=80) on adults and 56.9% (n=37) on the elderly 17 .

In Oliveira study the early diet for patients with gastrectomy was accepted in 74% of cases, with low incidence of gastrointestinal symptoms. In 2011 Jo regards acceptance by 89% and only 8% with gastrointestinal symptoms 20 approximately 96.9% in this study, the improvement in acceptance may be due to slight variation of the protocol (restrict-liquid, complete-liquid and pasty-liquid) 25 . It is noteworthy that the good thing is that these patients received caloric intake as early as possible, which also referenced by Hirao, where early diet group received higher caloric intake 3.2 days earlier than usual 12 .

Studies evaluating patients undergoing gastrectomy for gastric cancer claim that oral feeding on the first postoperative day is safe 20 19 .

Early feeding after operations involving resection and intestinal anastomosis can be conducted without risks and benefits as early discharge, lower incidence of infectious complications and lower hospital costs 1 , as well as Nakeeb shows that started early diet and got shorter hospital stay and less time to flatus, with good tolerance by patients (75%) 24 .

Regarding vomiting - which did not occur in this study - it is controversial; was present in 13% in Nascimento (2002) paper, related to intestinal anastomoses 4 .

Another symptom found more frequently was constipation, taken after a period of over three days without stool. Approximately 65.2% of patients were constipated postoperatively, but it must be considered the preoperative fasting time and approximately 48 h postoperatively and therefore no sufficient training of waste to evacuate, even with starting the diet. Another possible cause is the decrease in intestinal transit caused by the use of anesthetics, opioids and impaired mobility postoperatively.

The hospital stay was 5±2.2 days in these patients. According to Hur it was 8.03±1.43 days in early feeding group and 9.9±2 in the control group 14 . According to Jeong, hospitalization period was 7.4 days in early feeding group and 8.9 days in the control group 19 . Nascimento that evaluated early feeding after intestinal anastomoses, shows 10 days to the early group and 12 to control 4 . It can be seen that early feeding group has shorter hospital length of stay both in patients with gastrectomy and in other operations with intestinal anastomoses. While there is no comparison between the early group and the control group in this study, the length of stay was lower compared with the other two studies cited above 14 19 . This difference may be attributed to the year in which these studies were conducted because, from then on, surgical techniques and nutritional therapy were improved.

## CONCLUSION

Early feeding in total or partial gastrectomy postoperative period was well tolerated by patients.
